# Fish Macrophages Show Distinct Metabolic Signatures Upon Polarization

**DOI:** 10.3389/fimmu.2020.00152

**Published:** 2020-02-25

**Authors:** Annelieke S. Wentzel, Joëlle J. E. Janssen, Vincent C. J. de Boer, Wouter G. van Veen, Maria Forlenza, Geert F. Wiegertjes

**Affiliations:** ^1^Cell Biology and Immunology Group, Wageningen University & Research, Wageningen, Netherlands; ^2^Human and Animal Physiology Group, Wageningen University & Research, Wageningen, Netherlands; ^3^Experimental Zoology Group, Wageningen University & Research, Wageningen, Netherlands; ^4^Aquaculture and Fisheries Group, Wageningen University & Research, Wageningen, Netherlands

**Keywords:** M1 M2 macrophage polarization, metabolic reprogramming, teleost, glycolysis, oxidative phosphorylation (OXPHOS), oxidative metabolism, Seahorse, extracellular flux analysis

## Abstract

Macrophages play important roles in conditions ranging from host immune defense to tissue regeneration and polarize their functional phenotype accordingly. Next to differences in the use of L-arginine and the production of different cytokines, inflammatory M1 macrophages and anti-inflammatory M2 macrophages are also metabolically distinct. In mammals, M1 macrophages show metabolic reprogramming toward glycolysis, while M2 macrophages rely on oxidative phosphorylation to generate energy. The presence of polarized functional immune phenotypes conserved from mammals to fish led us to hypothesize that a similar metabolic reprogramming in polarized macrophages exists in carp. We studied mitochondrial function of M1 and M2 carp macrophages under basal and stressed conditions to determine oxidative capacity by real-time measurements of oxygen consumption and glycolytic capacity by measuring lactate-based acidification. In M1 macrophages, we found increased nitric oxide production and *irg1* expression in addition to altered oxidative phosphorylation and glycolysis. In M2 macrophages, we found increased arginase activity, and both oxidative phosphorylation and glycolysis were similar to control macrophages. These results indicate that M1 and M2 carp macrophages show distinct metabolic signatures and indicate that metabolic reprogramming may occur in carp M1 macrophages. This immunometabolic reprogramming likely supports the inflammatory phenotype of polarized macrophages in teleost fish such as carp, similar to what has been shown in mammals.

## Introduction

Macrophages are essential innate immune cells involved in host defense that play a role in initiating inflammation but also play a role in the resolution phase of inflammation and in tissue regeneration. These opposing conditions provide microenvironments that drive innate immune cells such as macrophages to display specific effector functions and tailor immune response to either combat pathogens or repair damage. In mammals, depending on the exact microenvironment, an array of different macrophage phenotypes can exist, with the most polarized phenotypes termed M1 and M2 (1). Inflammatory macrophages are commonly associated with T helper-1 responses (hence M1) and produce pro-inflammatory cytokines, antimicrobial nitric oxide (NO), or other reactive oxygen radicals (ROS) (2–4). Anti-inflammatory macrophages are commonly associated with T helper-2 responses (hence M2), produce anti-inflammatory cytokines, and show increased arginase activity. Hence, M1 macrophages metabolize the amino acid L-arginine to produce NO, while M2 macrophages metabolize the same substrate to produce proline and polyamines (3). Thus, M1 and M2 macrophages show opposing metabolism of L-arginine.

**Figure 1:**
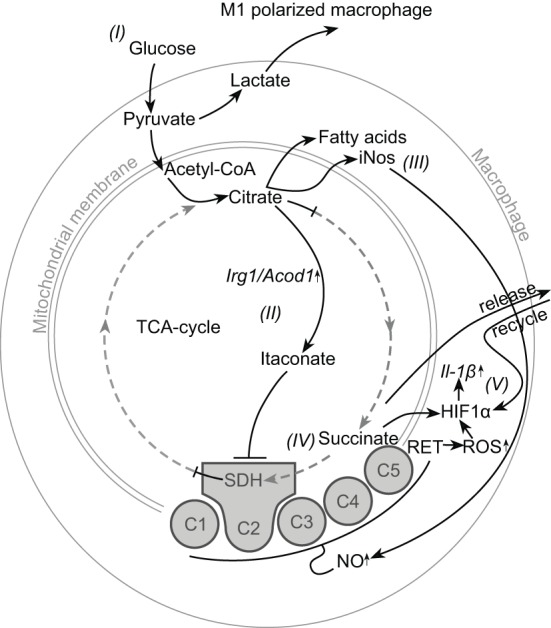
Schematic representation of metabolic reprogramming toward glycolysis in M1 macrophages. Pathways indicated in black are retained or enhanced in M1, pathways indicated in (dashed) gray are reduced in M1. The uptake of glucose and glycolysis (I) is increased in M1, which results in excess pyruvate that is converted to lactate and released from the cell. In the mitochondria, two breaks in the TCA cycle result in the accumulation of citrate and succinate. Accumulation of citrate contributes to three important changes: (1) Citrate is converted to itaconate by the enzyme encoded by *Irg1* (II). Itaconate in turn can inhibit both the TCA cycle and the electron transport chain by blocking succinate dehydrogenase (SDH). (2) Citrate is also shuttled out of the mitochondria, where it promotes NO production and fatty acid synthesis (III). (3) Oxidative phosphorylation through the electron transport chain is inhibited, both by itaconate-mediated inhibition of SDH and by increased production of NO (IV). This causes increased levels of succinate, which stabilize hypoxia-inducible factor 1-alpha (HIF1α) and are also released and recycled by the cell. ROS generated by reverse electron transport (RET) over the hyperpolarized mitochondrial membrane also stabilize HIF1α, which in turn results in increased Il-1β expression (V). Figure based on (6).

In mammals, macrophages are also metabolically reprogrammed to enhance opposing pathways to generate energy upon polarization [reviewed by (5, 6)]. Most studies addressing macrophage immunometabolism have been performed in mice. IL-4–activated M2 macrophages rely primarily on oxidative phosphorylation (OXPHOS) for energy production, with the exact role of fatty acid oxidation still being debated (6). In contrast, upon activation with bacterial lipopolysaccharide (LPS) alone or in combination with IFN-γ, M1 macrophages show metabolic reprogramming from OXPHOS toward glycolysis. Reprogramming of M1 macrophages toward glycolysis is accompanied by two “breaks” in the tricarboxylic acid cycle (TCA cycle) and inhibition of parts of the electron transport chain (ETC) in the mitochondria (5) (Figure 1). The two breaks in the TCA cycle are due to lower activity and expression of isocitrate dehydrogenase and succinate dehydrogenase (SDH) and lead to an accumulation of citrate and succinate (Figure 1), which supports important pro-inflammatory immune functions of M1 macrophages. For example, accumulated citrate is shuttled out of the mitochondria, and subsequent accumulation in the cytosol contributes to the production of NO, ROS, and fatty acid synthesis for membrane and granule formation. Accumulated succinate contributes to ROS production and can stabilize hypoxia-inducible factor 1-alpha (HIF1α), which activates the glycolytic pathway and drives inflammation through increased expression of IL-1β (7). Released succinate acts as an alarmin in the extracellular microenvironment and is recycled to generate a feed-forward loop, further increasing IL-1β production (8). Last but not least, inhibition of the ETC is mediated both by NO and itaconate (Figure 1). Itaconate, produced from citrate with the enzyme encoded by *irg1*, is considered an important regulator of metabolic reprogramming, as it inhibits both the ETC and TCA cycle through SDH, but is also important to dampen inflammatory functions at later time points (6, 9). Therefore, metabolic reprogramming from oxidative metabolism to glycolysis supports several inflammatory immune functions in M1 macrophages.

Fish macrophages show several of the immune functions typically associated with M1 and M2 macrophages, and thus, macrophage polarization may be largely conserved (10–12). For example, M1 macrophages of carp show increased NO production after stimulation with LPS alone (13, 14) or in combination with Ifn-γ (15) and show increased expression of *il-1*β (10, 14). Zebrafish macrophages show stabilization of Hif1α and *il-1*β expression following mycobacterial infection (16, 17). M2 macrophages of carp and goldfish show increased arginase activity after stimulation with cAMP or Il-4 (10, 18). The apparent conservation of macrophage polarization led us to hypothesize a conservation of the underlying changes in energy metabolism and immunometabolic reprogramming in fish macrophages. We therefore studied mitochondrial function of M1 and M2 polarized carp macrophages under basal and stressed conditions. We determined oxidative capacity by real-time measurements of oxygen consumption, and we measured glycolytic capacity by measuring lactate-based acidification. Our data provide the first evidence that carp macrophages can use different pathways for energy metabolism associated with macrophage polarization in teleost fish. We discuss the implications of our findings for studying macrophage polarization in exothermic aquatic vertebrates.

## Materials and Methods

### Animals

European common carp (*Cyprinus carpio carpio* L.) used for experiments were the offspring of a cross between the R3 strain of Polish origin and the R8 strain of Hungarian origin (19). Carp were bred and reared in the aquatic research facility of Wageningen University and Research at 20–23°C in recirculating UV-treated tap water and fed pelleted dry food (Skretting, Nutreco) twice daily. All experiments were performed with the approval of the Animal Experiments Committee of Wageningen University and Research (Ethical Committee documentation number 2017.W-0034).

### *In vitro* Culture and Polarization of Head Kidney–Derived Carp Macrophages

The head kidney in teleost fish is a primary hematopoietic organ and can be considered the functional equivalent of bone marrow (20). Head kidney–derived macrophages (from hereon referred to as macrophages) were obtained as previously described (10). After 6 days of culture at 27°C, macrophages were polarized to M1 or M2 state. In short, macrophages were harvested by gentle scraping after incubation on ice for 15 min. Cells were pelleted at 450 × g for 10 min at 4°C before resuspension in cRPMI+ [RPMI 1640 culture medium with 25 mM HEPES and 2 mM L-glutamine (12-115F, Lonza), supplemented with L-glutamine (2 mM, Gibco), penicillin G (100 U/ml), streptomycin sulfate (100 μg/ml, Gibco) and heat-inactivated pooled carp serum (1.5% v/v)]. Cells were cultured at 27°C in the presence of 5% CO_2_ in cRPMI+ unless indicated otherwise. Macrophages stimulated for 24 h with 20 or 50 μg/ml LPS (*Escherichia coli*, L2880, Sigma-Aldrich) were considered M1. Macrophages stimulated for 24 h with 0.5 mg/ml dibutyryl cAMP (N^6^,2′-O-dibutryladenosine 3′:5′-cyclic monophosphate sodium D0627, Sigma Aldrich, abbreviated as cAMP) were considered M2.

### NO Production

NO production for confirmation of functional polarization was determined in culture supernatants of polarized macrophages. In brief, 5 × 10^5^ macrophages per well were seeded in 96-well plates (Corning) in 150 μl of cRPMI+. After polarization, NO production was determined as nitrite in 75 μl culture supernatant as described previously (21) and expressed in μM using a nitrite standard curve.

### Arginase Activity

Arginase enzymatic activity for confirmation of functional polarization into M2 was measured in cell lysates and normalized using a ratio of the sample protein content compared to lysate of control cells. A total of 1.5 × 10^6^ cells polarized for 24 h in 450 μl cRPMI+ were lysed in 100 μl of 0.1% Triton X-100. Protein content of the samples was determined using the Bradford protein dye reagent (Bio-Rad) according to the manufacturer's protocol. Arginase activity was measured in 25 μl lysate essentially as described previously for 50 μl lysate (10), but volumes were scaled down accordingly. Arginase activity was determined as the conversion of L-arginine to urea by arginase and expressed in nmol/min/10^6^ cells.

### Extracellular Lactate

The release of lactate into the culture supernatant was measured using a lactate colorimetric assay (Kit II K627, BioVision) in filtered samples (Amicon 10K spin column, Z677108-96EA, Sigma-Aldrich) according to the manufacturer's instructions. Briefly, 1.5 × 10^6^ cells were polarized in 450 μl cRPMI+ before culture supernatants from triplicate wells were pooled and filtered. Fifty microliters of 25 × diluted culture supernatant was combined with 50 μl reaction mix in a 96-well plate and incubated for 30 min at room temperature. OD was measured at 450 nm, and the concentration of lactate present in culture supernatants was calculated based on a calibration curve supplied by the manufacturer.

### Mito Stress Test

Extracellular flux analysis of polarized macrophages was performed by measuring oxygen consumption rate (OCR) and extracellular acidification rate (ECAR) using a Seahorse XFe96 extracellular flux analyzer (Agilent). We essentially applied the manufacturer's protocol and optimized culture conditions, cell density, and carbonyl cyanide-4 (trifluoromethoxy) phenylhydrazone (FCCP) concentrations to measure OCR and ECAR in carp macrophages and adjusted all incubation steps in the protocol to 27°C. For this, the XFe96 analyzer was kept at room temperature and set to 20°C, which would keep the analyzer at a stable 27°C ± 1°C during the complete assay.

To measure OCR and ECAR, culture medium of 1 × 10^5^ macrophages/well-polarized for 24 h in XF96 V3 PS Cell Culture Microplates (Agilent) was replaced with 180 μl non-buffered Seahorse XF base medium supplemented with 10 mM D-glucose (Sigma) and 4 mM L-glutamine (Gibco) at pH 7.4. After incubation without CO_2_ for 45 min at 27°C, OCR and ECAR were measured at basal level and after subsequent addition of 1.5 μM oligomycin, 0.2 μM FCCP, and 2.5 μM antimycin A/1.25 μM Rotenone/40 μM Hoechst DNA stain (all from Sigma). The standard 20 min equilibration cycle at the beginning of a Seahorse run was replaced by an incubation for 10 min without additional mixing before measurements were started. Measurement cycles consisted of 1 min mixing, 1 min waiting, and 3 min measuring. A minimum of four technical replicates were used for each condition.

To normalize OCR and ECAR measurements, we determined the area covered with Hoechst stained nuclei for each well according the manufacturer's instructions. We subsequently used the ratio for each well-compared to the average of all controls for normalization of the OCR and ECAR data. Images were taken with a Cytation 1 plate reader (BioTek) and analyzed using CellProfiler (Version 3.1.9).

### Real-Time Activation of Macrophages

To track glycolysis and oxidative metabolism during activation of macrophages in real time, 1 × 10^5^ macrophages/well were plated in XF96 V3 cell culture plates and cultured overnight. The cell culture medium was replaced with 180 μl Seahorse XF RPMI medium with 10 mM D-glucose and 4 mM L-glutamine (pH 7.4). After incubation without CO_2_ for 45 min, OCR and ECAR were recorded at basal level and for at least 4 h after addition of 20 or 50 μg/ml LPS, 0.5 mg/ml cAMP, or medium for unstimulated controls as the first injection in the Seahorse run. The standard 20 min equilibration cycle at the beginning of a Seahorse run was replaced by an incubation for 10 min without additional mixing. Measurement cycles consist of 30 s mixing, 1.5 min waiting, and 3 min measuring. A minimum of four technical replicates were used for each condition.

### Gene Expression Analysis of *irg1*

Transcriptome sequencing was performed as described previously (22, 23). After reads were aligned to the latest genome assembly of common carp (BioProject: PRJNA73579) (22), differential gene expression was analyzed using the bioinformatics package DESeq 2.0 (v1.22.2) and R statistical software (3.5.5) (24) as described before (23). Statistical analysis was performed using a paired design with unstimulated cells as control and performed for LPS-stimulated (30 μg/ml) and cAMP-stimulated (0.5 mg/ml) macrophages independently (*n* = 3 independent cultures for each stimulus).

### Statistics

The mean of technical replicates was used for paired statistical analysis of *n* = 6 biological replicates (NO production, arginase activity, and Mito Stress test), *n* = 5 biological replicates (lactate assay), or *n* = 3 biological replicates (gene expression).

Analysis of NO, arginase assays, and lactate assays was performed with a repeated-measures ANOVA followed by Tukey's *post hoc* tests to determine significant differences between treatments. Normal distributions were confirmed (Shapiro–Wilk test), and in the absence of sphericity (Mauchly's test of sphericity), the Greenhouse–Geisser correction was applied. For Mito Stress test analysis, Friedman's two-way ANOVA by ranks was used followed by Dunn's *post hoc* tests for the non-normally distributed samples. Statistical analysis was performed using IBM SPSS statistics Version 26 and GraphPad Prism 5. Gene expression analysis was performed using DESeq2 as described above. Differences were considered significant when *p* < 0.05.

## Results

### Metabolic Signatures of Polarized Carp Macrophages

**Figure 2:**
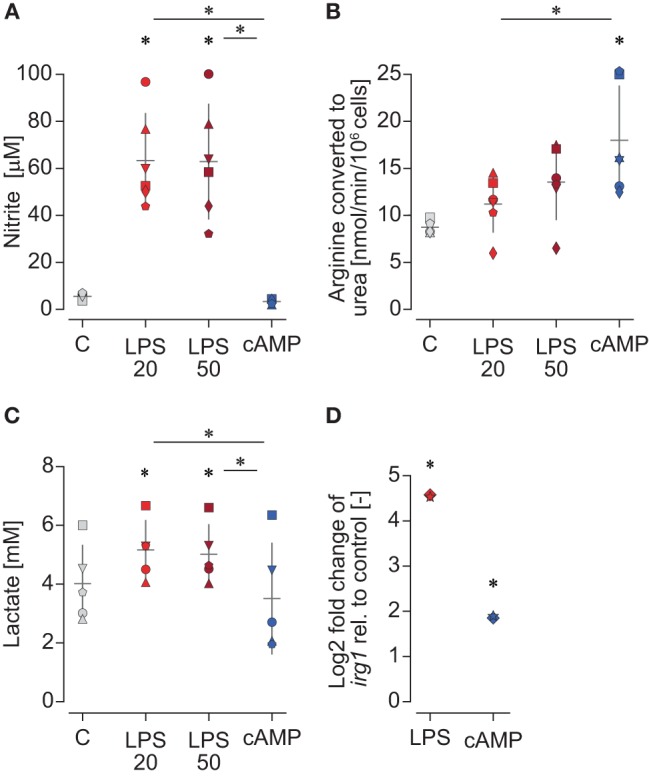
Polarized macrophages of carp show indications of distinct metabolic profiles. Carp macrophages were left unstimulated (gray, control) or were polarized for 24 h with LPS (red; 20 or 50 μg/ml) or with cyclic AMP (blue; 0.5 mg/ml). **(A)** Nitric oxide production measured as nitrite concentration (μM). **(B)** Arginase activity measured as conversion of L-arginine to urea by arginase (nmol/min/10^6^ cells). **(C)** Lactate concentration (mM). Shown are individual fish (each indicated by a unique symbol) and the mean and standard deviation of *n* = 6 **(A,B)** or *n* = 5 **(C)** biological replicates. **(D)** Gene expression of two carp *irg1* paralogs [cypCar_00026281 (star) and cypCar_00007903 (diamond)] stimulated for 6 h with 30 μg/ml LPS (red) or with 0.5 μg/ml cAMP (blue) analyzed by DEseq2 after transcriptome sequencing. Gene expression data are shown as log2 fold change compared to unstimulated controls (*n* = 3 biological replicates). Data **(A–C)** were analyzed using a repeated-measures ANOVA with Geisser–Greenhouse correction followed by Tukey's *post hoc* tests. Data **(D)** were analyzed by DEseq2 as part of a transcriptional study. Differences were considered significant when *p* < 0.05. Asterisks (*) indicate significant differences between stimulated and control groups or between groups (line with asterisk). Since there were no clear differences between the two concentrations of LPS, experiments were continued with 20 μg/ml LPS.

Macrophages were confirmed as polarized prior to determining their metabolic pathways. LPS-stimulated M1 macrophages showed increased NO production compared to unstimulated macrophages, while cAMP-stimulated macrophages did not (Figure 2A). cAMP-stimulated M2 macrophages showed increased arginase activity compared to unstimulated macrophages, while LPS-stimulated macrophages did not (Figure 2B).

Metabolic signatures of polarized carp macrophages were examined by measuring extracellular lactate production, expression of *irg1*, and accumulation of intracellular citrate and succinate. All these parameters were shown to play a role in the metabolic reprogramming of murine M1 macrophages from OXPHOS toward glycolysis. In carp macrophages, increased lactate concentrations were measured in culture supernatants of M1 but not M2 macrophages compared to unstimulated macrophages (Figure 2C). Also, gene expression of *irg1* was increased to a much higher extent in M1 than M2 macrophages (Figure 2D). Accumulation of intracellular citrate did not show differences between M1 and M2 macrophages, whereas intracellular succinate could not be quantified because levels were below the detection limit (data not shown). Overall, the combination of increased lactate production and increased *irg1* expression indicated that carp M1 macrophages showed a metabolic reprogramming toward glycolysis.

### OCR and ECAR of Polarized Carp Macrophages

**Figure 3:**
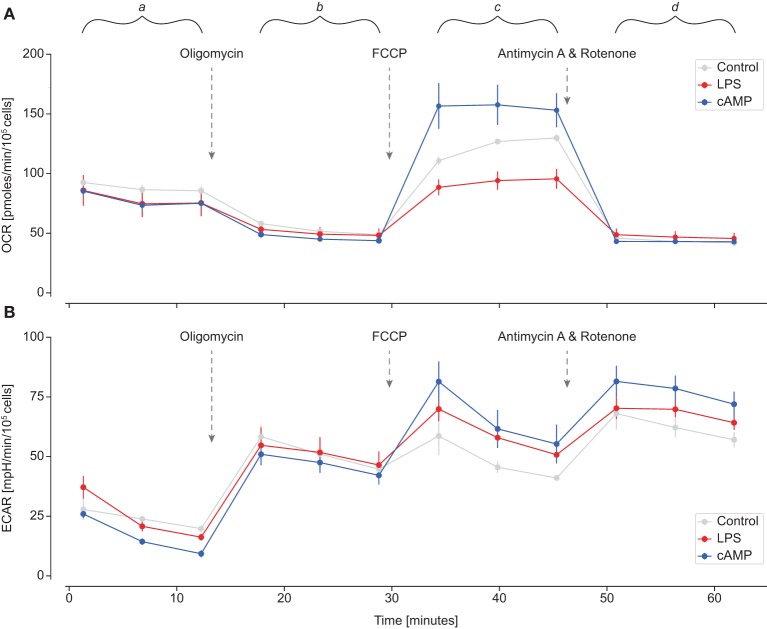
Oxygen consumption rates (OCRs) and extracellular acidification rate (ECAR) of polarized carp macrophages. Carp macrophages were left unstimulated (gray, control) or were polarized for 24 h with LPS (red; 20 μg/ml) or with cyclic AMP (blue; 0.5 μg/ml). Graphs display Mito Stress test profiles of **(A)** OCR and **(B)** ECAR at basal level (time range a) and after subsequent addition of oligomycin (time range b), carbonyl cyanide-4 (trifluoromethoxy) phenylhydrazone (FCCP) (time range c), and antimycin/rotenone (time range d). Normalized rates are shown as mean and SEM of one representative experiment out of *n* = 6 biological replicates.

To study in detail mitochondrial function and oxidative capacity in polarized carp macrophages, we first optimized the Seahorse Mito Stress test for use with carp macrophages at a lower (27°C) temperature. We optimized cell density to 1 × 10^5^ cells/well and found carp macrophages to be particularly sensitive to FCCP with a relatively low optimum concentration of 0.2 μM (tested range 0.1–3 μM). Then, we determined OCR (Figure 3A) as a measure for oxidative metabolism and ECAR (Figure 3B) as a measure for glycolysis. M1 and M2 macrophages did not show clear differences in OCR or ECAR at basal level (time range *a*). Injection of oligomycin blocks complex V of the ETC and as such inhibits ATP production. Both M1 and M2 macrophages therefore decreased oxygen consumption while increasing extracellular acidification (time range *b*). Disruption of the mitochondrial membrane potential by injection of FCCP induces maximal oxygen consumption. Indeed, after FCCP injection, M1 and M2 macrophages both increased oxygen consumption, but M1 macrophages clearly showed much lower OCR than control or M2 macrophages (time range *c*). Finally, injection of antimycin A and rotenone inhibits complex IIV and complex I of the ETC, thereby completely blocking the ETC. M1 and M2 macrophages did not show differences in non-mitochondrial respiration after antimycin A and rotenone were injected (time range *d*).

**Figure 4:**
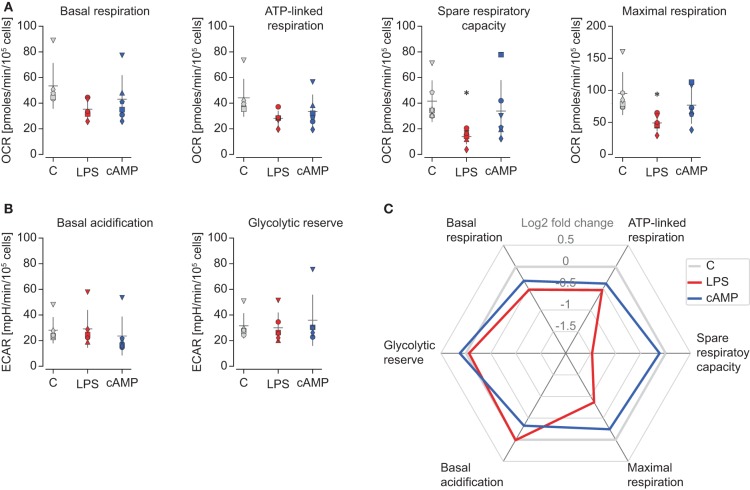
Metabolic parameters underline differences in oxidative potential between polarized carp macrophages. Carp macrophages were left unstimulated (gray, control) or were polarized for 24 h with LPS (red; 20 μg/ml) or with cyclic AMP (blue; 0.5 mg/ml). **(A)** Oxidative parameters based on OCR include basal respiration (OCR_a_–OCR_d_), oxygen used for ATP synthesis (OCR_a_–OCR_b_), maximal respiration (OCR_c_–OCR_d_), and spare respiratory capacity (OCR_c_–OCR_a_). **(B)** Glycolytic parameters based on ECAR include basal acidification rate (ECAR_a_) and glycolytic reserve (ECAR_b_–ECAR_a_). **(C)** Spider plot depicting both oxidative and glycolytic parameters of polarized carp macrophages (mean log2 fold change compared to respective controls). Metabolic parameters were calculated from normalized Mito Stress test profiles of polarized macrophages and based on the mean of three consecutive measurements as indicated in Figure 3 with time periods a, b, c, or d. Normalized rates are shown for individual fish (each indicated by a unique icon) and the mean and SD of *n* = 6 biological replicates. Differences were considered significant when *p* < 0.05. Asterisks (*) indicate significant differences between stimulated and control groups.

Oxygen consumption and extracellular acidification data were used to quantify different metabolic parameters. Basal respiration and ATP-linked respiration (OCR used for ATP synthesis) were not significantly different between control and polarized carp macrophages. However, spare respiratory capacity after injection with FCCP (time range c) was significantly impaired in M1 carp macrophages, which reflected the impaired capacity of M1 macrophages to increase respiration and meet increased energy demands when stressed. Maximal respiration was therefore also significantly reduced in M1 carp macrophages (Figure 4A). In contrast, basal acidification or glycolytic reserve did not change with polarization (Figure 4B), indicating that the above-discussed reduction in oxidative capacity of M1 was not mirrored by an increase in glycolysis. Taking all parameters together, polarized M1 macrophages of carp clearly show a different metabolic profile compared to control and M2 macrophages (Figure 4C).

**Figure 5:**
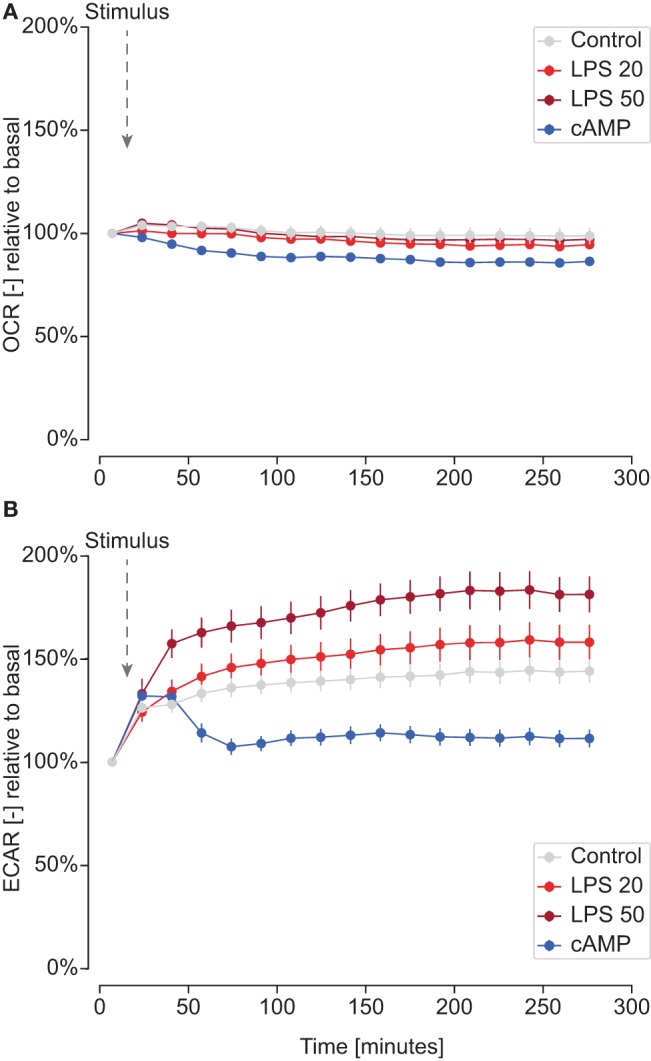
Real-time measurements of ECAR after activation with LPS or cyclic AMP. Carp macrophages were left unstimulated (gray, control) or were stimulated with LPS (red; 20 μg/ml or 50 μg/ml) or with cyclic AMP (blue; 0.5 mg/ml) by injection after determining basal OCR and ECAR levels. Panels represent real-time measurements of OCR **(A)** and ECAR **(B)** for one representative fish out of two. Means of three consecutive OCR and ECAR measurements were normalized to basal level for each well using the mean of three basal measurements before injection of the stimulus. Markers represents the mean and SEM of technical replicates expressed relative to basal rates.

Although lactate levels were increased in M1 macrophage culture supernatants (see Figure 2C), polarized carp macrophages did not show differences in basal ECAR or in glycolytic reserve after 24 h of polarization (see Figures 3B, 4B). This could indicate that ECAR normalized after 24 h to control ECAR levels and that ECAR peaked at earlier time points than 24 h. We thus performed a preliminary real-time measurement of OCR and ECAR before, during, and immediately after activation of carp macrophages. We observed a rapid, dose-dependent increase in ECAR that remained high for the duration of the experiment, but only in M1 macrophages (LPS stimulation) (Figure 5B). In contrast, M2 macrophages (cAMP stimulation) showed a rapid but very short increase in ECAR which rapidly returned to values below controls. No differences in OCR were observed within this time frame (Figure 5A). These results suggest that M1 carp rapidly increase their basal glycolysis and that this increase is sustained up to 4 h but reverts to basal levels at 24 h.

## Discussion

Previous studies have shown a general conservation of carp macrophage immune function with respect to their ability to polarize toward a pro- or anti-inflammatory profile in response to conventional M1 or M2 stimuli. These observations led us to hypothesize the occurrence of metabolic reprogramming of polarized macrophages of carp. To study this hypothesis, we determined the oxidative and glycolytic capacity of M1 and M2 carp macrophages by measuring OCRs and ECARs under basal and stressed conditions in real time. Carp M1 macrophages show (i) reduced maximal respiration and (ii) reduced spare respiratory capacity, both indicative of a reduction in oxidative capacity. Furthermore, carp M1 macrophages show (iii) increased lactate production after activation with LPS and a rapid increase in ECAR which is sustained up to 4 h but not 24 h. Finally, carp macrophages show (iv) increased production of NO and (v) increased gene expression of *irg1*, which encodes an enzyme that converts citrate to itaconate. Itaconate is a metabolite that can inhibit both the TCA cycle and the ETC, thus contributing to reduced oxidative capacity. Overall, carp M1 but not M2 macrophages show reduced oxidative metabolism and increased glycolysis.

To date, immunometabolic reprogramming of polarized macrophages has been demonstrated primarily in mice, where polarized macrophages show opposing pathways for energy metabolism: M2s rely on OXPHOS, whereas M1s are metabolically reprogrammed toward glycolysis. Our results indicate that carp M1 macrophages alter their energy metabolism in a manner similar to what has been described for murine M1 macrophages. On the other hand, carp M2 macrophages did not significantly alter their energy metabolism from control cells. Using real-time measurements similar to the ones applied in the present study for carp, M1 murine macrophages were shown to reprogram their energy metabolism toward glycolysis (25–27).

**Figure 6:**
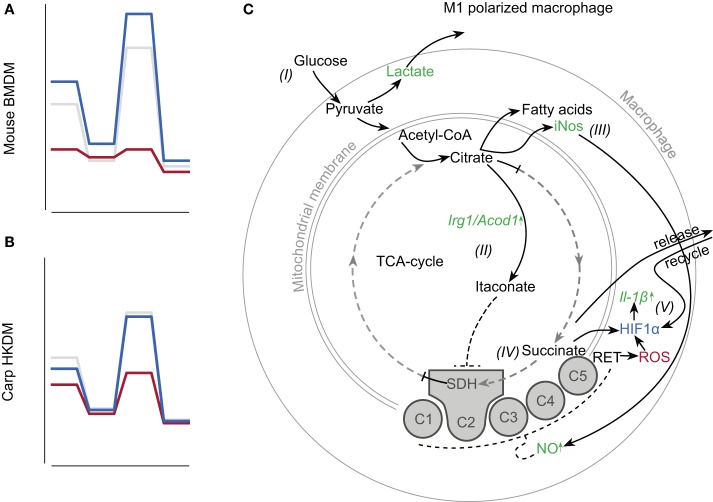
Schematic representation of metabolic reprogramming of carp M1 macrophages upon stimulation with LPS. Schematics of OCRs for **(A)** murine bone marrow–derived macrophages [BMDMs; based on (25, 26)] and **(B)** carp head kidney–derived macrophages (HKDMs; this study). Colors represent control macrophages (gray), polarized M1 macrophages (red), or M2 macrophages (blue). **(C)** Schematic representation of metabolic reprogramming toward glycolysis in murine macrophages, but modified for carp M1 macrophages. Pathways in black are enhanced and pathways in gray with dashed lines are decreased in murine M1 macrophages. Text in green refers to intermediates with regulation in LPS-stimulated carp macrophages similar to regulation in mice. Text in blue refers to intermediates present in zebrafish but not yet studied in carp. Text in red refers to mechanisms present in carp M1 macrophages but not regulated similarly to those in mice. Figure based on (6). Description: (I) Increased lactate in culture supernatants suggests increased glycolysis. (II) LPS-stimulated macrophages show increased *irg1* expression and (III) increased *inos* gene expression and NO production. (IV) Reduced oxidative capacity suggests inhibition of the ETC to some degree, but the mechanism needs confirmation. Succinate accumulation could not be confirmed at this point. (V) Hif1α stabilization exists under inflammatory conditions and is linked to increased *il-1*β transcription in zebrafish (16, 17). Increased *il-1*β expression has been shown in carp (14), but causation has yet to be determined. ROS production occurs in carp macrophages but is low after LPS stimulation (28). Both succinate and ROS can theoretically stabilize Hif-1α, but the mechanisms need to be confirmed for carp.

At basal level, murine M1 macrophages show increased glycolysis and reduced OXPHOS. When pushed toward maximal capacity, murine M1 macrophages show a drastic decrease in maximal respiration and spare respiratory capacity. This metabolic reprogramming appears to be responsible for their inability to repolarize from M1 to M2, as they do not regain their oxidative capacity upon repolarization, whereas M2 can repolarize into M1 macrophages without problems (26). At basal level, carp M1 macrophages did not show the increased glycolysis and reduced OXPHOS observed for murine M1 macrophages. This could be because the initial reprogramming of carp LPS-stimulated macrophages toward glycolysis had already been normalized at the start of our measurements. The absence of differences at basal level could be the result of several differences in experimental circumstances between the studies on macrophages of mouse and carp, among which are the exact origin of macrophages, stimuli, and temperature. However, the absence of a difference at basal level may also suggest that carp M1 macrophages were not terminally differentiated by LPS and could possibly still repolarize from M1 to M2, a hypothesis of interest for future studies. Overall, and similar to what has been observed for murine macrophages, carp M1 macrophages show reduced oxidative capacity when pushed to maximal respiration (Figures 6A,B). Although the absolute difference between polarized M1 and M2 macrophages appears smaller in carp than in mice, the energy metabolism of carp M1 macrophages appears similar to that of murine M1 macrophages.

In this study, we gained important insights into the metabolic pathways used by carp M1 macrophages and compared these to the metabolic pathways described for M1 polarized macrophages of mice (Figure 6C). Carp M1 macrophages increase lactate production and shift toward glycolysis immediately after stimulation with LPS, although the exact kinetics remain to be studied. Although we could not detect differences between M1 and M2 in citrate accumulation, we did detect an upregulation of *irg1* expression, which potentially leads to increased itaconate. In mice, both itaconate and NO can contribute to an inhibition of the ETC. Although we can detect increased *inos* gene expression and increased production of NO in carp M1 macrophages, the contribution of itaconate and/or NO to inhibition of the ETC in carp macrophages remains to be studied. Furthermore, although we previously reported an upregulation of *il-1*β in macrophages stimulated with LPS (10, 14) and although it is known that Hif1α is stabilized and linked to *il-1*β expression during mycobacterium infection of zebrafish (16, 17), it remains to be confirmed if Hif1α stabilization is required for *il-1*β expression in carp. Since we do not generally observe ROS production by carp macrophages in response to LPS (28) and were not able to measure intracellular succinate, it remains to be determined which of the two would contribute to the stabilization of Hif1α. Overall, we provide evidence of clear similarities as well as differences between polarized macrophages of mouse and carp.

Carp M2 macrophages did not show a clear increase in maximal respiration compared to controls. Moreover, differences between basal and maximal capacity appeared to be relatively small when compared to those of mice (26). Again, differences in experimental conditions between the studies on macrophages of mouse and carp, among which are the exact origin of macrophages, stimuli, and temperature, can maybe help explain such differences. However, respiration in carp macrophages may also be regulated within more narrow boundaries than in mice: controlled use of oxygen may be particularly important in animals that breathe under water, where available oxygen levels can be more often critical than in air. Studies into the effect of oxygen availability on cellular energy metabolism, in particular, the metabolic reprogramming of innate immune cells, may therefore be of high interest for aquatic animals. Furthermore, oxygen availability is inversely related to temperature (29), and temperature can also directly influence mitochondrial function. For example, at lower temperatures, composition of the mitochondrial membrane changes to counteract reduced membrane fluidity, which in turn changes the ADP affinity of the mitochondria [reviewed by (30)]. Temperature may thus play an important role in metabolic reprogramming. Carp are ectothermic fish that can be acclimatized to a large temperature range and a large range of oxygen pressures, which makes our model adaptable to study mitochondrial functioning and metabolic reprogramming of innate immune cells under varying environmental conditions.

Our studies confirm the general conservation of carp macrophage immune function with respect to their ability to polarize toward a pro- or anti-inflammatory profile in response to conventional M1 or M2 stimuli, and further studies could refine the extent of this conservation. Our studies also help to improve the understanding of fundamental mechanisms underlying energy metabolism and metabolic reprogramming of immune cells in teleost fish and open a field of comparative immunometabolism for exothermics aquatic vertebrates.

## Data Availability Statement

The datasets generated for this study are available on request to the corresponding author.

## Ethics Statement

The animal study was reviewed and approved by Animal Experiments Committee of Wageningen University and Research.

## Author Contributions

AW, JJ, VB, and GW contributed to the design of the experiments. AW performed experiments, and AW, WV, MF, and GW contributed to the analysis of data. GW acquired funding. AW drafted the manuscript, and GW, WV, MF, JJ, and VB critically reviewed the manuscript.

### Conflict of Interest

The authors declare that the research was conducted in the absence of any commercial or financial relationships that could be construed as a potential conflict of interest.

## Abbreviations

ADP: adenosine diphosphate
ATP: adenosine triphosphate
BMDM: bone marrow–derived macrophage
cAMP: N^6^,2′-O-dibutryladenosine 3′:5′-cyclic monophosphate sodium
ECAR: extracellular acidification rate
ETC: electron transport chain
FCCP: carbonyl cyanide-4 (trifluoromethoxy) phenylhydrazone
HIF1α: hypoxia-inducible factor 1-alpha
HKDM: head kidney–derived macrophage
IFN-γ: interferon-gamma
IL-1β: interleukin 1-beta
IL-4: interleukin 4
Irg1/Acod1: immune responsive gene 1/aconitate decarboxylase 1
LPS: lipopolysaccharide
NO: nitric oxide
OCR: oxygen consumption rate
OXPHOS: oxidative phosphorylation
PCS: pooled carp serum
RET: reverse electron transport
ROS: reactive oxygen species
SDH: succinate dehydrogenase
TCA cycle: tricarboxylic acid cycle.
